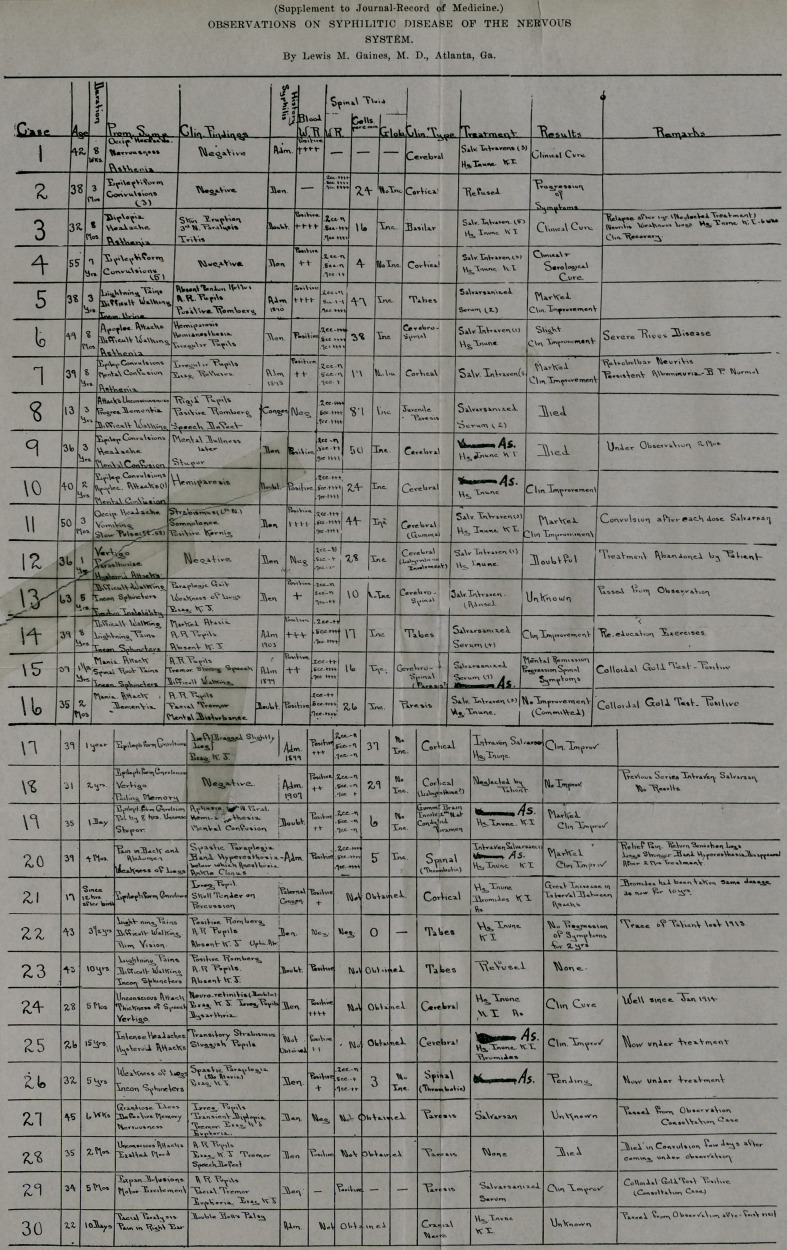# Observation on Syphilitic Disease of the Nervous System

**Published:** 1915-08

**Authors:** Lewis M. Gaines

**Affiliations:** Atlanta, Ga.; Associate Professor of Medicine (Mental Diseases and Clinical Neurology) Emory University School of Medicine, Atlanta, Ga. 1023 Empire Building.


### OBSERVATIONS ON SYPHILITIC DISEASE OF THE NERVOUS SYSTEM.

By Lewis M. Gaines, M. D., Atlanta, Ga.

Associate Professor of Medicine (Mental Diseases and Clinical Neurology) Emory University School of Medicine, Atlanta, Ga.

Syphilis is the most potent cause of diseased conditions of the nervous system. Using an algebraic mode of expression we may say: Many diseases of the nervous system = syphilis + x. The combination of various predisposing causes, the majority of which are problematical or at best theoretical, with syphilis, originates various forms of disease of the nervous system to which we have given various names for the sake of convenience. It must be said, however, that the majority of these diseases were described and recognized many years before we realized the important role syphilis has played in the causation.

Assuming that the Wassermann reaction, accurately performed, and completely interpreted in connection with definite clinical symptoms, is indicative of the presence of syphilis, we may say that syphilis is found to be the important causative factor in a very considerable number of patients presenting quite diverse clinical symptoms. There is no symptom or group of symptoms diagnostic of syphilis of the nervous system though there are many which are extremely suggestive. The most striking single feature of syphilitic disease of the nervous system is the extraordinary diversity of clinical symptoms presented by a series of cases.

Syphilitic disease of the nervous system can no longer be considered a rare condition. One must now consider under the head of syphilitic diseases, tabes, general paresis, many varying types of cerebrospinal syphilis, many cases of epilepsy, many cases of celebral arterial disease, many cases of paraplegia, and many cases of peripheral nerve disease. Prior to our refinement of diagnosis, syphilis was frequently unsuspected.

As would be expected the variety of pathological changes found, corresponds to the diversity of clinical symptoms. Arterial changes, meningitis, myelitis, degenerations of tracts and nerve cells, pathological exudates, and later sclerotic changes in various locations in brain and cord give rise to innumerable clinical pictures. These pathological changes are the result of long continued activity on the part of the spirochaetae. In this connection it is of interest and importance to refer to the recent work of Wile and Stokes on the involvement of the nervous system during the primary stage of syphilis. These investigators found from their study of the spinal fluid that the nervous system in at least sixty to seventy per cent. of cases is involved before there are other evidences of the hematogenous spread of the spirochaetae from the site of the initial lesion. Such involvement may be slight and nonproductive of clinical symptoms, or may be serious and early. It would appear from the study of these observers that the central nervous system is one of the most attractive places in the body for the spirochaetae. Whether or not clinical symptoms are produced would appear to depend upon the unknown factor which I have called x. Furthermore the spirochaetae may lie dormant in the central nervous system over a period of time varying from a few weeks to thirty or more years. The conclusion is inevitable that any one with uncured syphilis is liable at any time to develop syphilitic disease of the nervous system depending upon the operation of predisposing causes concerning the nature of which we are at present comparatively ignorant.

It becomes a matter of the greatest importance to ascertain in a given case whether or not syphilis is the cause. Briefly the diagnosis must rest upon, first, the clinical history and, second, the result of laboratory findings. In regard to the clinical history denial of infection is of no value. The presence of certain symptoms is suspicious. Such symptoms are epipleptiform attacks occurring after the age of thirty, epipleptiform attacks with rapid recovery especially where high blood pressure and nephritis can be excluded, involvement of various cranial nerves especially the second, third, and eighth, persistent headaches, abnormalities of the deep reflexes, many types of motor disturbances in the limbs, and needless to say any of the classical signs of tabes or general paresis. The list is not complete, but I believe embraces the most striking suggestive symptoms.

The laboratory occupies a very necessary place in the examination. We must weigh clinical evidence and the laboratory findings together, not separately. A Wassermann should be done on the blood. If positive, assuming we believe in the reliability of the test and in the competence of the man who does the test, syphilis is present. Whether or not it is a case of syphilis of the nervous system depends upon the clinical findings. If the blood Wassermann is negative it by no means proves the absence of syphilis. Our next task is to examine the spinal fluid. Many of my series of cases have shown a persistently negative Wassermann on the blood with positive spinal fluid findings. In examining the spinal fluid one desires a Wassermann test, a cell count, a globulin estimation, and in certain cases Lange's colloidal gold reaction. A positive spinal fluid Wassermann, a cell count of more than ten lymphocytes per cubic millimeter and in some cases an increase in globulins means syphilis involving the nervous system.

The accompanying chart indicates the principal features in the series of cases now reported.

How shall we treat patients with syphilitic disease of the nervous system? Although we have comparatively few remedies, there are many methods of applying them. It would lead us too far afield to discuss in detail various methods of treatment. From my experience I find it very valuable to institute a definite plan of treatment at the outset. First, the patient is given a series of intravenous injections of Salvarsan, the dose of which depends upon the individual case. The length of time between injections varies from one to two weeks. In connection with Salvarsan the patient is given mercury, preferably by inunction, or by hypodermic administration which is, however, painful and therefore discouraging to the patient. I am impressed with the inefficacy of mercury by mouth. Careful directions must be given to the patient concerning personal hygiene, diet and habits. The use of potassium iodide is frequently of doubtful benefit contrary to long established precedent. Sometimes its use appears to be actually harmful. If results are not forthcoming as shown by abatement of clinical symptoms and more favorable laboratory reports one should consider the use of intraspinal injections of salvarsanized serum after the method of Swift and Ellis. This method seems to be of particular benefit in Tabes. Two cases in my series had not improved under salvarsan administered in other ways.

(Supplement to Journal-Record of Medicine.)

### OBSERVATIONS ON SYPHILITIC DISEASE OF THE NERVOUS SYSTEM.

In conclusion, there is no doubt concerning the frequency of syphilitic disease of the nervous system, their non-recognition in many cases, the many disguises under which they masquerade, the failure to relieve them unless the cause is appreciated, and the brilliant results which frequently may be obtained, provided they are recognized early and treated thoroughly and systematically.

1023 Empire Building.

## Figures and Tables

**Figure f1:**